# Enhancing Functional Properties of Edible Films through the Combined Ultrasound-Assisted Extraction and Cold Plasma Treatment of Moroccan Pumpkin Seeds (*Cucurbita pepo*) Protein Isolates

**DOI:** 10.1021/acsomega.5c02566

**Published:** 2025-07-11

**Authors:** Walid Zenasni, Hande Yenipazar, Said Ennahli, Aadil Bajoub, Celale Kırkın, Rachid Nejjari, Hanaa Abdelmoumen, Neşe Şahin Yeşilçubuk, El Amine Ajal

**Affiliations:** † Laboratory of Food and Food Byproducts Chemistry and Processing Technology, National School of Agriculture in Meknès, km 10, Haj Kaddour Road, B.P. S/40, Meknès, Morocco; ‡ UPR of Pharmacognosy, Faculty of Medicine and Pharmacy of Rabat, 107736 Mohammed V University, B.P. 6203, Rabat 10000, Morocco; § Faculty of Sciences Rabat, Mohammed V University, Rabat, BP 1014 RP, Morocco; ∥ Department of Food Engineering, Faculty of Chemical and Metallurgical Engineering, 52971 İstanbul Technical University, 34469 Maslak, İstanbul, Türkiye

## Abstract

This study evaluates the effectiveness of combining ultrasound-assisted extraction and cold plasma treatment to improve the mechanical, barrier, and functional properties of edible films made from Moroccan pumpkin seed protein isolates. Ultrasound-assisted extraction was conducted at 20 kHz and 45–50% power with a 1:10 ratio of pumpkin seed sample to alkali solution for 30 min. The process yielded a protein-rich isolate (87.10%) with high solubility at pH 9 and good absorption capacities for water (1.80 ± 0.06 mL/g) and fat (3.88 ± 0.08 mL/g) and a gelling capacity of 12%. The films produced from these isolates demonstrated promising functional properties for food packaging applications. Further improvement of the film properties was achieved through cold plasma treatment using a 40 kV pulsed DC power supply at 56 kHz for 0, 10, 20, and 30 min. This treatment stabilized the moisture content (10.79–11.16%) and significantly reduced swelling (from 204.87% to 159.09%), suggesting enhanced barrier properties that could extend food shelf life. Although transparency decreased slightly (from 0.94 to 1.20), the films retained acceptable visibility and exhibited promising ultraviolet barrier properties, making them suitable for packaging light-sensitive food products.

## Graphical abstract

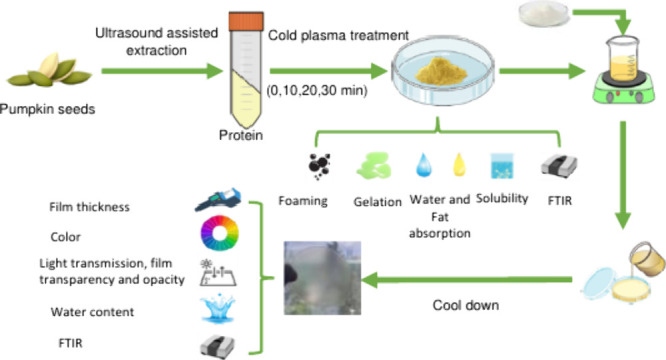

## 1 Introduction

In the current economic context, where there is a growing awareness of environmental issues and increasing pressure on actors in the agri-food sector to adopt more sustainable practices, the use of plastics for food packaging poses a major challenge.^1^ While plastic has been favored for its convenience and relatively low cost, its impact on the environment is becoming increasingly threatening.^2^ These nonbiodegradable materials, contribute to marine, soil and atmospheric pollution and pose a significant threat to wildlife.^3^ This problem is particularly pressing in a global context where resources for plastic production are decreasing and the demand for a circular and sustainable economy in the agri-food sector is increasing.^4^ Therefore, the search for alternatives to plastics that both optimize the use of natural resources and improve the properties of packaging has become central to global efforts to develop sustainable circular economy in the agri-food sector.^5,6^

When it comes to tackling the environmental challenges associated with food packaging, natural-based polymer films derived from some components of food waste or byproducts of food processing are proving to be promising solutions.^4^ Unlike conventional plastics, these biobased films are nontoxic, renewable, and biodegradable, and their production process has a lower carbon footprint,^7,8^ making them a more sustainable alternative to reduce the use of plastics and minimize the environmental impact of the agri-food sector.^9^

Among the various ingredients derived from food waste and byproducts of the agri-food industry, proteins stand out due to their potential in the development of environmentally friendly and functional packaging materials. Research has shown that these substances possess outstanding film-forming capabilities, and offer both structural integrity and nutritional benefits. The different amino acid profiles of the proteins improve the intermolecular binding properties, which significantly enhances the functional characteristics of the resulting biofilms.^10^ The latter typically exhibit remarkable transparency, elasticity, plasticity, and effective oxygen barrier properties, as reported by several studies,^11,12^ making them highly suitable for various packaging applications.

Within the promising protein sources, pumpkin seeds, one of the main byproducts of pumpkin processing, prove to be particularly valuable.^13^ Whole pumpkin seeds contain between 24% and 36% protein, while defatted seeds can reach up to 54%.^14^ This high protein content not only meets the amino acid standards set by the Food and Agriculture Organization (FAO) but, according to recent research, also offers a nontoxic, sustainable alternative to synthetic food packaging materials. Studies have shown that the structural characteristics of pumpkin seed globulin proteins are similar to those of legume seeds, suggesting similar bioavailability and functional properties when used in bioedible films.^15,16^ Therefore, the use of pumpkin seed proteins for the development of biobased materials, such as edible films and coatings, could bring significant environmental and economic benefits to the agri-food and food packaging industries.^17^

In Morocco, cultivation covers a considerable area of around 11,570 ha, with an average annual production of around 329,177 tons.^18^ This level of production highlights the potential for the valorization of pumpkin byproducts, especially the seeds, for new applications in the food and packaging sector. However, while pumpkin seed protein is promising for the development of biofilms, its recovery poses a major challenge, especially during the extraction step.^19^ In the commonly used alkaline extraction method, the proteins are dissolved in an alkaline solution and then precipitated at their isoelectric point.^20^ This approach requires harsh conditions such as high pH values and temperatures as well as long extraction times, which can lead to protein denaturation.^21^ As a result, this process often leads to limited extraction yields and compromises the functional properties of the final protein isolates.^22^ In addition, the high energy consumption and environmental impact of the method have raised concerns about its sustainability and overall efficiency.^23^

To address these challenges, researchers are exploring alternative techniques to improve both the yield and quality of proteins extracted from pumpkin seeds. Given the thermal sensitivity of these nutrients, there is a growing focus on nonthermal processing technologies as viable alternatives to alkaline extraction methods.^24^ In this regard, ultrasound-assisted extraction is gaining attention as a promising approach.^25,26^ This technique employs ultrasonic waves to generate cavitation, which enhances solvent penetration into the plant matrix and facilitates protein release.^23,27^ Consequently, ultrasound-assisted extraction has the potential to boost proteins yield while better preserving their functional properties, making them more suitable for use in biofilm formulations.^21^

Alongside ultrasound-assisted extraction, cold plasma technology is gaining recognition for its potential to enhance the functionality of protein-based films.^28^ This nonthermal technique not only improves the safety and quality of protein isolates but also avoids chemical residues, making it an environmentally friendly alternative.^29^ Furthermore, numerous recent studies underscore the benefits of this technology for protein films. Specifically, it has been demonstrated that cold plasma treatment induces cross-linking within protein structures, resulting in more cohesive and denser biofilms.^30–32^ This cross-linking effect improves the mechanical strength and durability of the films, making them more suitable for applications like food packaging and biodegradable coatings.^28,33^ Additionally, the cold plasma treatment enhances the hydrophobicity and barrier properties of protein films, broadening their potential applications across various industries.^30^

Considering the advantages offered by both techniques, it is anticipated that combining ultrasound-assisted extraction with subsequent cold plasma treatment of protein isolates could address the challenges associated with improving the physicochemical properties of the proteins and the food films derived from them. Integrating these methods could represent a significant advance in the development of high-quality, functional protein-based materials. These innovative approaches not only overcome the limitations of conventional extraction techniques but also offer sustainable and efficient solutions for the production of biofilms with improved properties.

Against this background, the present study investigates the potential of integrating these two techniques for the extraction and valorization of pumpkin seed proteins, focusing on the development of food biofilms. The aim is to drive the development of protein-based materials and promote more sustainable practices in the agri-food and packaging sectors, both in Morocco and internationally.

## 2 Results and Discussion

### 2.1 Characterization of Pumpkin Seed Protein Isolate (PSPI)

#### 2.1.1 Protein Yield and Content of PSPI

The protein content of the defatted pumpkin seed was 51.89%, which aligns with previous studies by.^14,23^ Following UAE, there was a significant increase of 35.21% in protein, resulting in a protein content of 87.1 ± 1.47% in the PSPI and a yield of 43.20 ± 0.85% (Table 1). These findings closely align with those reported by,^23^ who found a protein content of 86.07% and a yield of 48.91%. The observed increase in protein content underscores the effectiveness of UAE in maximizing protein yield from pumpkin seeds. Numerous studies report a significant improvement in protein extraction yield when ultrasonic treatment is applied, either as a pretreatment or during the extraction process. Increases of 10% to over 100% have been observed compared to conventional methods.^34^ Additionally, the mechanical and cavitation effects induced by UAE likely contributed to the enhanced extraction efficiency, as demonstrated in studies on the extraction of proteins of quinoa, black bean, and lentil^35^ and soybean.^36,37^ Indeed, it seemed that localized high temperatures and pressures improves the solubility and release of proteins while facilitating the movement of proteins from the solid matrix to the solvent providing enhanced mass transfer.^38,39^ These findings highlight the potential of UAE as a valuable tool for protein extraction, offering opportunities for improving protein yields and enhancing functionality in various food and industrial applications.

**Table 1:** Properties of Pumpkin Seeds Protein Isolate for Producing Edible Films^a^

**Parameter**	**Value**
**Protein Content (PSPI)**	87.10 ± 1.47%
**Protein Extraction Yield**	43.20 ± 0.85%
**Water Absorption Capacity (WAC)**	1.80 ± 0.06 mL/g
**Fat Absorption Capacity (FAC)**	3.88 ± 0.08 mL/g
**Foam Capacity (FC)**	100%
**Foam Stability (FS)**	57.14%
**Least Gelation Concentration (LGC)**	12%

a All data are the mean ± SD of three replicates.

#### 2.1.2 Protein Solubility

Protein solubility is a fundamental techno-functional criterion as relevant properties are directly determined by the extent of hydration and aqueous solubilization.^40^ PSPI solubility exhibits a strong pH dependence with an inverse bell-shaped curve across a pH range of 2–10. (Figure 1). The lowest protein solubility values for all samples occurred at pH 4 and 5, which can be attributed to the protein aggregation and precipitation at the isoelectric point. At this point, proteins are electrically neutral and molecules tend to bind and precipitate, resulting in lower solubility.^41,42^ Previous studies reported the same trend in different plant proteins.^43–52^ Because of the high solubility of PSPI at acidic pH values (2–3), they can be incorporated into edible biopackaging films designed for the packaging of acidic foods. Such films would offer potential advantages due to their intrinsic antimicrobial properties associated with the low pH environment, and the high protein content of PSPI films could contribute to improved barrier properties and mechanical strength, thereby enhancing their overall functionality.

**Figure 1:**
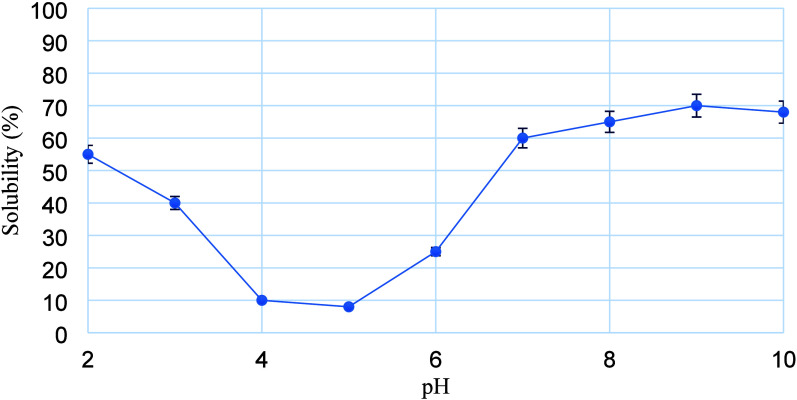
Solubility of pumpkin seeds protein isolate.

#### 2.1.3 Water Absorption Capacity and Fat Absorption Capacity

Understanding the water absorption capacity (WAC) and fat absorption capacity (FAC) of a protein is crucial for predicting its impact on texture, mouthfeel, and sensory attributes in food applications.^53,54^ In this study, PSPI exhibited a WAC of 1.80 ± 0.06 mL/g (Table 1), suggesting their potential to enhance juiciness, tenderness, texture, emulsion stability, and even foam stability in food products.^53^ This high WAC may be partially attributed to the presence of dietary fiber, a known contributor to water binding.^55^ PSPI also demonstrated a substantial FAC of 3.88 ± 0.08 mL/g, indicating potential benefits for fat and flavor retention, as well as controlled oil release.^54^

These values of WAC and FAC were higher than those reported by^23^ (1.14 g/g and 1.20 g/g, respectively), but comparable to those of another study^50^ (1.29–1.35 mL/g for WAC and 3.59–3.70 mL/g for FAC). This suggests that PSPI’s superior absorption capacities could be influenced by both its fiber content and its amino acid composition, which favors strong lipid binding.^23,41,53^ Furthermore, the FAC of PSPI in this study surpassed that of other plant protein sources like quinoa isolates,^56^ soy protein isolates,^54^ flaxseed protein concentrate,^57^ sour cherry kernel protein concentrate,^58^ and chickpea protein concentrate.^59^

#### 2.1.4 Foaming Capacity and Stability

The foaming capacity (FC) and stability (FS) of PSPI are critical functional properties, impacting its performance in various food applications. FC reflects the protein’s ability to create a foam upon agitation with air or water, while FS determines the foam’s persistence over time. These properties act as important indices of the whipping potential of a protein isolate.^60^ In this study, PSPI exhibited an FC of 100%, more than those reported for grass peas and soybeans (41% and 24–36%, respectively).^61,62^ However, its FS of 57.14% was slightly lower than those observed for the same plant protein sources (62% and 68%, respectively). The high FC of PSPI can be attributed to its protein solubility at neutral and alkaline pH values. Foaming performance is impacted by protein solubility.^63–65^ Increased protein–water interactions at these pH ranges facilitate protein structure unfolding and enhance air encapsulation, leading to greater initial foam volume.^27^ Furthermore, the distribution of protein sizes within a source influences its foaming properties.^63,66^

#### 2.1.5 Least Gelation Concentration

The gel-forming characteristics of plant proteins are essential for developing semisolid textures in various food applications.^67^ Assessing this gelling property often relies on the least gelation concentration (LGC), which represents the minimum protein concentration needed to form a gel that maintains its shape and resists flow when inverted.^68^ Lower LGC values indicate superior gelation ability, highlighting the protein’s potential for creating firm textures.^67^ In this study, PSPI exhibited an LGC of 12%. While this suggests good gelation potential, gels formed at lower concentrations (11% and 10%) displayed softer textures, indicating a concentration-dependent effect on gel strength. Furthermore, the samples at 9% and 8% protein concentrations became highly viscous, highlighting the versatility of PSPI across a range of potential applications. The least gelation concentration of most plant proteins falls within the range of 10–18%, but some of them can form gels at considerably lower concentrations. For instance, chickpea proteins have an LGC value of around 5–7%. It should be noted that the reported LGC values depend on gelation conditions, such as pH, ionic strength, and heating conditions, as well as on protein type and the presence of other ingredients. Consequently, the same protein may have different LGC values depending on the experimental conditions, highlighting the importance of standardizing methodologies when comparing different protein sources.

#### 2.1.6 FTIR Analysis

Fourier-transform infrared (FTIR) spectroscopy was used to analyze the molecular structure of PSPI. The recorded spectra reveal distinct peaks at various wavenumbers, each indicative of specific molecular vibrations and functional groups (Figure 2). The results, shed light on the potential molecular constituents of the PSPI. The peak at 3278 cm^–1^, the wavenumber is associated with O–H and N–H stretching vibrations, suggesting the possible presence of water or hydroxyl groups,^69^ the peak at 3062 cm^–1^ represents C–H stretching vibrations.^70^

**Figure 2:**
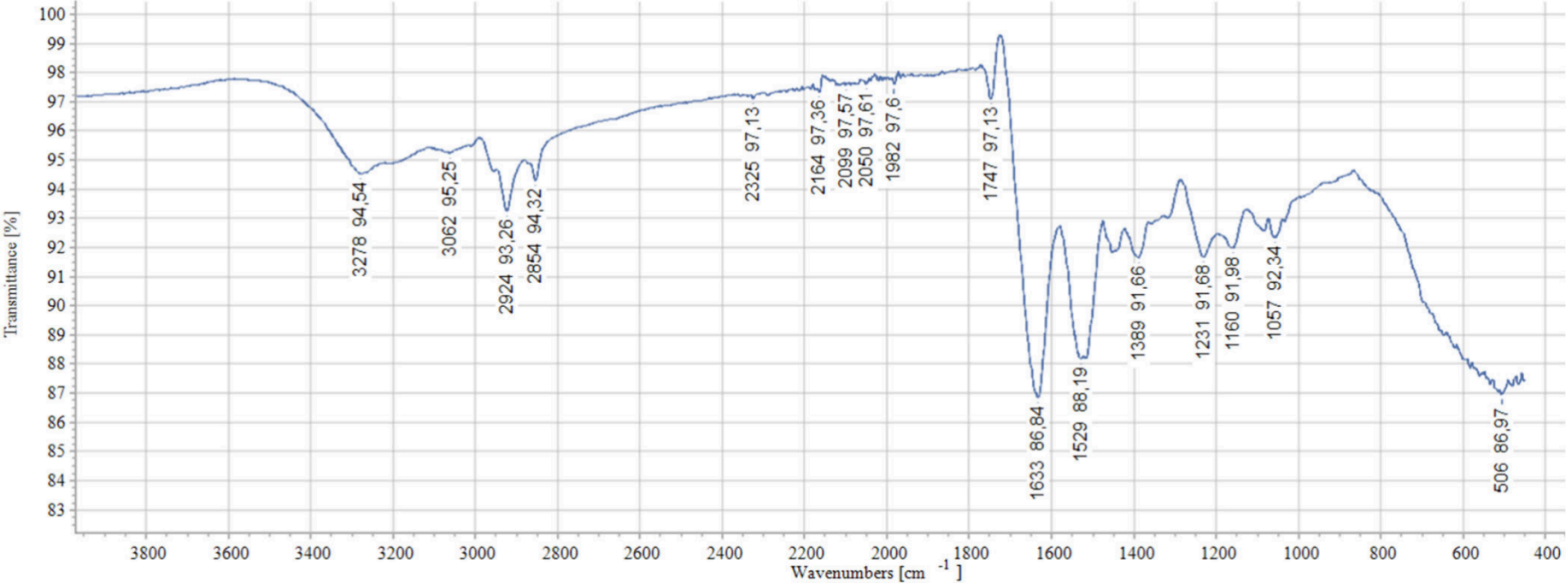
FTIR spectrum protein isolate of pumpkin seeds.

While at 2924 cm^–1^ and 2854 cm^–1^, correspond to C–H stretching vibrations in aliphatic compounds, indicating the presence of aliphatic hydrocarbons. The peaks at 2325 cm^–1^, 2164 cm^–1^, 2099.2 cm^–1^, and 1982 cm^–1^ correspond to amide I and amide II bands suggesting the presence of proteins. The specific wavenumbers hint at different protein structures within the isolate.^68^ The peak at 1747 cm^–1^ indicates C=O stretching vibrations, potentially linked to ester carbonyl groups. This may signify the presence of lipids or other carbonyl-containing compounds. For 1633 cm^–1^, the wavenumber indicates the amide I band, associated with C=O stretching vibrations in proteins. The presence of the amide II band at 1529 cm^–1^ is indicative of N–H bending and C–N stretching in proteins. Various functional groups in proteins at 1389 cm^–1^, 1231 cm^–1^, 1160 cm^–1^, and 1057 cm^–1^, are potentially related to amino acid side chain vibrations. The peak at 506 cm^–1^ is located in the lower wavenumber region, possibly indicating the presence of large molecular structures or specific functional groups.^71–77^

### 2.2 Characterization of Edible Films

#### 2.2.1 Film Preparation

After drying in the oven, the edible films were removed and found to be intact with a smooth surface (Figure 3). However, some undissolved components were present, suggesting that PSPI may not have fully dissolved or dispersed uniformly in the solution before film casting. These undissolved particles could lead to a textured or grainy appearance in the final film. Protein denaturation via heat, acid, base, or solvents causes proteins to unfold and expose reactive groups, initiating intermolecular bonding through various interactions to form networks or films, with the extent of bonding influenced by the degree of denaturation and amino acid composition. Adjusting pH and selecting the type and concentration of dissociated salts allows for the control of protein electrostatic and ionic conformation, and further modification can prevent agglomeration and ensure suitable film formation.^78^ Characteristics of the obtained film samples were given in Table 2.

**Figure 3:**
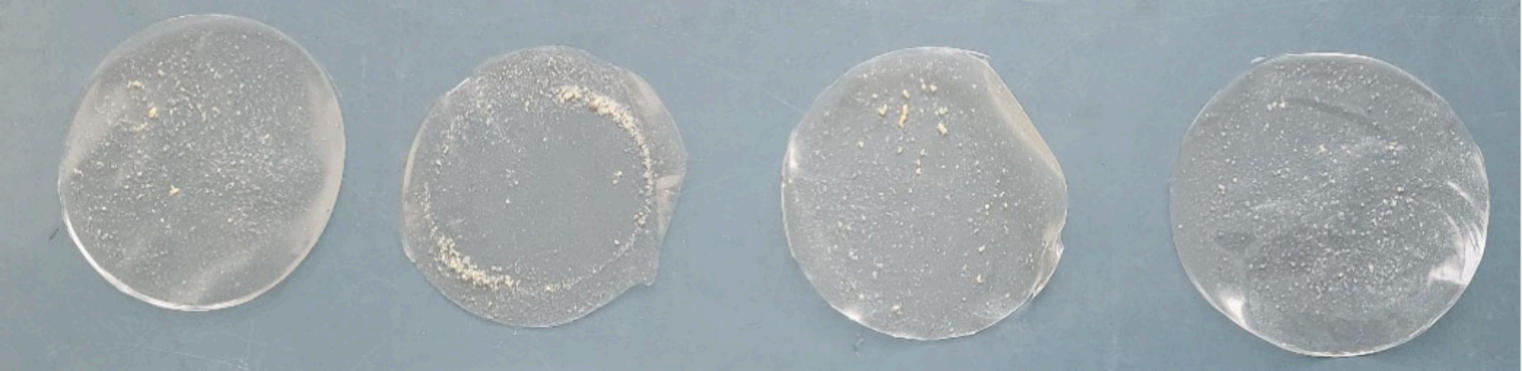
PSPI biodegradable films (from left to right F0, F10, F20, F30).

**Table 2:** Properties of Edible Films from Isolated Protein Treated with Cold Plasma^a^

**Film no** ^b^	**Water content (%)**	**Degree of swelling (%)**	**Water solubility (%)**	**Thickness (mm)**	**L** ^b^	**a** ^b^	**b** ^b^	**Transparency**
**F0**	10.79 ± 1.01 a	204.87 ± 6.92 a	7.31 ± 0.02 b	0.087 ± 0.005 a	43.64 ± 0.15 a	1.47 ± 0.04 a	1.42 ± 0.02 ab	0.94 ± 0.02 a
**F10**	10.80 ± 1.02 a	191.89 ± 5.77 ab	7.84 ± 0.01 ab	0.052 ± 0.006 b	42.72 ± 0.19 a	1.61 ± 0.05 b	1.50 ± 0.05 b	1.03 ± 0.02 b
**F20**	11.16 ± 0.33 a	170.30 ± 1.61 bc	8.00 ± 0.56 ab	0.093 ± 0.005a	43.49 ± 0.17 a	1.48 ± 0.02 a	1.29 ± 0.05 a	1.04 ± 0.02ab
**F30**	11.00 ± 0.58 a	159.09 ± 8.08 c	9.05 ± 0.01 a	0.092 ± 0.007 a	43.57 ± 0.18 a	1.49 ± 0.02 a	1.27 ± 0.04 a	1.20 ± 0.02ab

a Data are presented as mean ± SD (*n* = 3). Within columns, means with different letters are significantly different (*p* ≤ 0.05).

b F0, F10, F20, and F30 represent films prepared from protein isolates treated with varying levels of cold plasma.

#### 2.2.2 Water Content

The moisture content of the cold plasma-treated PSPI films was measured to assess their water-bonding capacity.^79,80^ Across all tested films, including those treated with cold plasma for (10, 20, and 30 min) and without cold plasma treatment, the moisture content ranged between 10.79% and 11.18% (Table 2). No significant difference in the moisture content was observed between the treated samples and the control (F0), suggesting that the cold plasma treatment did not alter the films’ moisture absorption capacity.^81^ Moreover, the observed moisture content values were lower than those reported for films made from other biopolymers, indicating strong moisture barrier properties.^82^ However, a notable increase in the water-holding capacity (WHC) of myofibrillar proteins from 21.8% in the control to 92.6% following cold plasma treatment^83^ has been demonstrated. They proposed that surface alterations may have facilitated protein unfolding, allowing for the formation of cross-links between protein strands. These cross-links, in turn, could trap significant amounts of water, leading to the observed enhancement in WHC. Moreover, it is reported that treating peanut isolated protein with plasma generated by a dielectric barrier discharge system led to alterations in its secondary structure and resulted in higher water-holding capacity.^81^ The impact of ultrasound on the techno-functional properties of proteins, particularly their water-holding capacity, has been demonstrated in various studies. For instance, ultrasound treatment of a millet protein concentrate solution improved its water-holding capacity.^82^ The discrepancy in the effect of UAE on water-holding capacity between pumpkin seed protein isolates (PSPI) and other protein sources may be due to differences in protein structure and composition. Variations in protein conformation, molecular weight, and surface characteristics could influence the response to ultrasonic treatment. Additionally, specific conditions of ultrasound treatment, such as intensity, frequency, and duration, may also play a role. Further research is needed to understand the factors contributing to these differences in water-holding capacity between PSPI and other protein sources under UAE conditions.

#### 2.2.3 Degree of Swelling, Water Solubility, and Thickness

The water-related characteristics of edible films, including swelling degree and water solubility, play a critical role in various food packaging applications.^84^ The application of cold plasma treatment to pumpkin seed protein isolate (PSPI) films resulted in a significant reduction in swelling, with values decreasing from 204.87 ± 6.92 (F0, control) to 159.09 ± 8.08 (F30) (Table 2). This aligns with previous studies reporting a significant reduction in swelling after cold plasma treatment.^28,85^

The reduced swelling observed in the treated films likely stems from several mechanisms. First, excessive exposure to cold plasma may induce modifications in the protein structure, altering its conformation and potentially concealing hydrophilic groups, thus limiting water uptake. Additionally, cross-linking induced by cold plasma could lead to the formation of a denser and more rigid film structure, reducing the available space for water absorption.^86^ Furthermore, depending on the plasma gas or processing conditions utilized, there may be an introduction of hydrophobic moieties onto the film surface, resulting in increased surface hydrophobicity and repelling water, thereby further restricting its interaction with the underlying protein matrix.^28^

On the other hand, a small increase in water solubility was observed after the cold plasma treatment as shown in Table 2, protein films retained their stability during 24 h in water with no significant difference between the control and treated samples. The PSPI film thickness averaged as 0.09 ± 0.006 mm, with the exception of F10, which was measured as 0.052 ± 0.006 mm.

#### 2.2.4 Color Measurement

The colorimetric analysis showed that the L*, a*, and b* values of all PSPI films ranged consistently from 42.72 ± 0.19 to 43.64 ± 0.15, 1.47 ± 0.04 to 1.61 ± 0.05, and 1.27 ± 0.04 to 1.50 ± 0.05 respectively with significant differences observed for all color parameters (*p* ≤ 0.05). The observed L* values indicate a medium-dark shade of the films, while a* suggests a subtle red tint, albeit barely discernible, and b* reflects a slight shift toward yellow compared to a neutral color. Similarly, comparable findings were reported on pea protein isolate edible films.^87^ Specifically, the lightness (L*) of the pea protein isolate edible films remained consistent across the different treatment durations, with no significant differences observed. However, there was a reduction in yellowness (b*) initially observed at 30 s of exposure.^87^ It can be concluded that color changes in cold plasma treated samples are generally insignificant for treatments under five minutes, but are influenced by plasma parameters, such as, input power, voltage, carrier gas, and exposure time.^88^

#### 2.2.5 Transmittance, Transparency and Opacity

Light transmittance (T%) shown in Figure 4, quantifies the ability of edible films to act as barriers against potentially photooxidative wavelengths. Several T% peaks within the visible spectrum (400–800 nm) indicate selective light absorption, while a general decreasing trend in transmittance across the spectrum is observed for all samples. A sharp rise in transmittance occurs between 400 and 600 nm, followed by a plateau. Interestingly, the untreated film (F0) exhibits slightly higher transmittance reaching a max of 92,31% at 630 nm compared to plasma-treated films. This suggests that cold plasma treatment reduces transmittance due to surface roughening and pore formation.^89^ The results confirm that cold plasma treatment enhances the UV barrier properties of the films.

**Figure 4:**
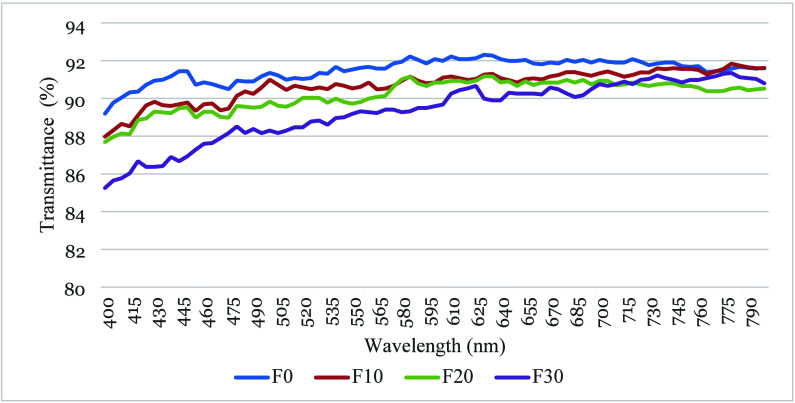
Transmittance (%) of edible films treated with cold plasma at wavelengths from 400 to 800 nm.

The transparency of the PSPI films at a wavelength of 600 nm is shown in Table 2. The values ranged from 0.94 to 1.20 and increased with higher treatment durations. The greater transparency value represents the lower transparency of the film, this means that F30 (1.2) is less transparent among all films, and in coherence with the Transmittance % results. The treated PSPI films let less light pass through due to their lower transparency. Results were in accordance with the results of soybean polysaccharide/gelatin blend edible films, which showed transparency ranging from 0.385 to 1.043.^90^ Based on the light transmittance and transparency data, all of the PSPI films were suitable for food packaging where product visibility is important.

On the other hand, opacity represents the film’s ability to block the passage of light and is crucial when using a film for food packaging or coating,^91^ low opacity values indicate a transparent edible film. Results represented in Figure 5 show that cold plasma increased the edible films’ opacity, same suggestion for transmittance can be approved for opacity, cold plasma can induce cross-linking within the polymerchains of the edible films.^89^

**Figure 5:**
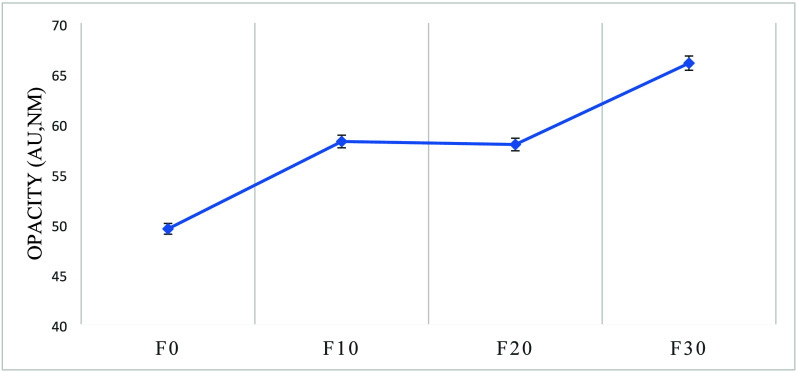
Opacity of edible films treated with cold plasma.

#### 2.2.6 FTIR Analysis of PSPI Films

The FTIR spectroscopy results, as shown in Figure 6, provide information about the molecular structure and composition of the PSPI films. First, all the samples exhibited peaks at ∼ 3000–3500 cm^–1^, which can be associated with water or free hydroxyl stretching bonds, and bonded OH groups of carboxylic acid.^92,93^ Furthermore, the peaks at ∼2800–3000 cm^–1^ indicated the methyl or methylene groups, C–H stretching vibrations in aliphatic compounds, symmetric and asymmetric stretching, and bending vibrations in the molecular structure.^93^ Moreover, 2349 cm^–1^ presenting amide bands suggest the presence of proteins. The stretching vibration of the conjugated peptide bond can be observed at 1636 cm^–1^. Polysaccharide-containing samples exhibit characteristic peaks between 800–1200 cm^–1^.^94^ Furthermore, the peak at 1371 cm^–1^ can be attributed to ester sulfate, while the bands between 1150 and 889 cm^–1^ can be attributed to the bridges formed by 3,6-anhydro-galactose.^95^ It is possible that the peak at 674 cm^–1^ reflects the presence of large molecular structures or specific functional groups in the lower wavenumber region, which indicates the presence of high molecular structures.

**Figure 6:**
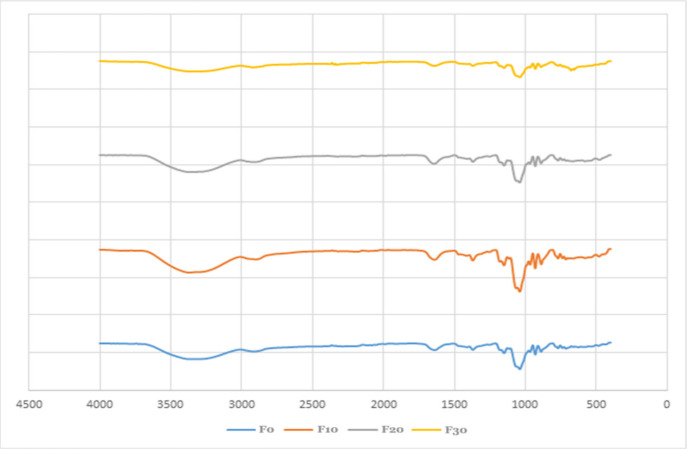
FTIR of PSPI films treated with cold plasma.

The intensities of the peaks decreased with prolonged cold plasma treatment, particularly in the F30 sample. Similar alterations following cold plasma treatment of packaging films were also reported by several authors.^96–98^ It can be concluded that cold plasma application alters the intensity of the obtained peaks, which may represent an increment of or interaction between functional groups.

## 3 Conclusion

This study highlights the promising potential of PSPI as a sustainable biopackaging material, in line with circular bioeconomy principles. Ultrasound-assisted extraction enhanced PSPI’s protein content, demonstrating its viability as a renewable packaging resource. The functional properties of PSPI films exhibited their versatility and suitability for various food packaging applications. Additionally, cold plasma treatment significantly improved the barrier properties of PSPI films, reducing moisture absorption and enhancing swelling behavior, albeit with minor changes in transparency. Despite this, PSPI films maintained acceptable product visibility and exhibited promising UV barrier properties, contributing to product stability and shelf life extension. Cold plasma treatment can be regarded as a key technique for precisely manipulating PSPI film characteristics, offering customizable and eco-friendly packaging solutions. Overall, this research underscores the importance of exploring biobased materials like PSPI for sustainable packaging practices, emphasizing their role in reducing environmental footprints and advancing the transition toward greener packaging alternatives.

## 4 Materials and Methods

### 4.1 Materials

In 2022, raw pumpkin (*Cucurbita peppo*) seeds were purchased from local farms and markets. Seeds were mixed evenly by weight to prepare a 3 kg composite sample.

All chemical reagents including N-hexane, sodium hydroxide, hydrochloric acid, agar, and reagents required for the Kjeldahl method were purchased from Sigma-Aldrich (St. Louis, MO, USA).

### 4.2 Preparation of the Defatted Pumpkin Seeds Samples

The raw seeds were processed following the method^14^ with slight modifications. The seeds were washed and manually cleaned to remove impurities and foreign materials before being dried at 45 °C for a period of 48 h. The dried seeds were ground into a fine powder using a 500 μm inox sieve and defatted with *n*-hexane (10:1, v/w) under continuous stirring. The mixture was centrifuged at 5000 rpm for 30 min to separate the solid fraction. The defatted meal was dried in a laboratory oven at 45 °C for 8 h and stored at 4 °C until further use.^23^

### 4.3 Ultrasound-Assisted Protein Extraction

The protein extraction was carried out following the method with minor modifications.^23^ UAE was performed using a Sonoplus HD 2200 ultrasonic homogenizer (Bandelin, Germany) at 20 kHz frequency with an MS 72 probe. The defatted powder was mixed with an alkali solution (sodium hydroxide, 1N) in a 100 mL beaker with a ratio of 1:10, w/v. The probe was inserted for a maximum of 1 cm deep. The protein extraction process lasted 30 min using 4 cycles at a power ranging between 45 and 50% of the maximum capacity while maintaining the temperature and pH constant at 32 °C and 9.5, respectively. After centrifugation at 5.000 × g for 15 min at 4 °C, the supernatant was collected, and the precipitate was dissolved in 0.1 N NaOH for 1 h. The centrifugation process lasted for 10 min, and the resulting supernatant was combined with the previous one for further protein isolation. The combined supernatant was adjusted with 0.1 N HCl to pH 5 and the supernatant was centrifuged at 7000 × g for 25 min at 4 °C. The precipitate was freeze-dried to obtain the PSPI and stored in the freezer at – 20 °C until used. The protein content of the isolate was determined by the Kjeldahl method, and the protein yield (PY) was calculated as

1 $$\mathrm{PY}\;\left(\%\right)=\frac{\mathrm{weight}\;\left(g\right)\;\mathrm{of PSPI}}{\mathrm{weight}\;\left(g\right)\;\mathrm{of meal}}\times 100$$

### 4.4 Characterization of the PSPI

#### 4.4.1 Protein Solubility

Protein solubility was determined by the method with minor modifications.^99^ 100 mg of the PSPI was mixed in 10 mL of deionized water to obtain protein dispersions. Then, the pH of the solutions was carefully adjusted from pH 2 to 10 with either HCl or NaOH with pH readjustment every 10 min. Protein contents in the solution before centrifugation and in the supernatants after centrifugation at 5000 g for 30 min were determined by a Kjeldahl method.^100^ Protein solubility was assessed by analyzing the nitrogen content in the supernatant, which represents the portion of the protein that stayed dissolved in the solution at different pH values. This value is expressed as a percentage shown in the following formula:

2 $$\mathrm{PS}\;\left(\%\right)=\frac{\mathrm{Amount of nitrogen in the supernatant}}{\mathrm{Total amount of nitrogen in}\;100\;\mathrm{mg}}\times 100$$

#### 4.4.2 Water Absorption Capacity and Fat Absorption Capacity

Water absorption capacity (WAC) and fat absorption capacity (FAC) analyses were conducted.^101^ PSPI sample (1 g) was suspended in distilled water (WAC) or olive oil (FAC) at a ratio of 1:10 (w/v) and well mixed using a vortex for 5 min then followed centrifugation at 3260g for 30 min, the supernatant volume was measured. WAC and FAC were subsequently calculated by quantifying the amount of water or oil retained by the respective samples.

3 $$WAC\;\left(\frac{ml}{g}\right)=\frac{Initial\;water\;volume-Superna\tan t\;volume\;after\;the\;centrifugation}{Mass\;of\;the\;sample}$$

4 $$FAC\;\left(\frac{ml}{g}\right)=\frac{Initial\;oil\;volume-Superna\tan t\;volume\;after\;the\;centrifugation}{Mass\;of\;the\;sample}$$

#### 4.4.3 Foaming Capacity and Stability

Foam capacity (FC) and foam stability (FS) analyses were determined following the methodology^50^ with slight modifications. A 10 mL sample of protein solution (10 mg/mL) was subjected to homogenization for 5 min. Subsequently, the solution was transferred to a graduated cylinder, and the initial and final volumes were noted. FC was defined as the percentage increase in volume relative to the initial volume, while FS was assessed by monitoring the volumetric changes over 120 min of storage. Calculations of FC and FS were performed using the following equations:

5 $$\mathrm{FC}\;\left(\%\right)=\frac{\mathrm{Volume after stirring}-\mathrm{Volume before stirring}}{\mathrm{Volume before stirring}}\times 100$$

6 $$FS\;\left(\%\right)=\frac{Volume\;after\;120\;\mathrm{min of standing}-Volume\;before\;stirring}{Volume\;after\;stirring-Volume\;before\;stirring}\times 100$$

#### 4.4.5 Least Gelation Concentration

To determine the minimum (least) gelation concentration (LGC), PSPI solutions ranging from 1% to 20% (w/v) were prepared in 10 mL aliquots within individual test tubes. Following 5 min of vigorous mixing on a vortex mixer, each solution was heated in a boiling water bath for 1 h. Subsequently, the tubes were cooled in a cold room at 4 °C for 2 h. The LGC was determined as the lowest concentration at which the sample maintained its stability, showing no tendency to fall or slip from the inverted tube.^102^

#### 4.4.6 FTIR Analysis

The structural profiles of the samples were analyzed using Fourier-transform infrared (FTIR) spectroscopy in triplicate. Measurements were acquired on a Spectrum Two FT-IR spectrometer at a resolution of 4 cm ^–1^, scanning across the mid-infrared range from 4000 to 500 cm^–1^.

### 4.5 Preparation of Edible Films

#### 4.5.1 Cold Plasma Pretreatment

The cold plasma treatment of PSPI for edible film production was conducted using an Asentek Model (Asentek, Ankara, Türkiye) 40 kV pulsed DC high-voltage power supply (40 kV pulsed DC HVPS). In this system, one electrode is grounded, while the other receives high-voltage pulses (40 kV/56 kHz). One g of PSPI placed in Petri dishes underwent treatment at a frequency of 56 kHz for 0, 10, 20, and 30 min (F0, F10, F20, and F30). The samples were treated between two stainless steel electrodes (12 cm of diameter and 4 mm of thickness). The bottom electrode was coupled with a glass barrier (2 mm of thickness) on which the samples were placed. The gap between the glass barrier and the top electrode was 1.5 cm.

#### 4.5.2 Film Preparation

Edible films were prepared by mixing cold-plasma-treated isolated undried pumpkin protein and agar with a 0.65:0.85 ratio. First, 100 mL of distilled water was heated to 100 °C and homogenized after incorporation of the PSPI and agar mixture. Using the solvent casting method, 30 mL aliquots of each solution were poured into Petri dishes (8.7 cm diameter) and dried overnight at 55 °C.^103^

#### 4.5.3 Water Content, Degree of Swelling, and Water Solubility

Water content (WC), degree of swelling (DS), and water solubility (DS) were calculated,^103^ all PSPI edible films were cut into 2 × 2 cm then put for 24 h at room temperature in order to determine the water content (W0). Then, the initial dry weight (W1) was calculated by drying films for 24 h at 75 °C. After being placed on Petri dishes with 30 mL of distilled water inside, the films were kept for a day and samples were weighed (W2) after removing gently the surface water with a paper towel. Finally, films were dried for an additional 24 h at 30 °C to determine the final dry weight (W3). The following equations were used to determine the WC, DS, and WS based on the data collected:

7 $$\mathrm{WC}\;\left(\%\right)=\frac{W0-W1}{W0}\times 100$$

8 $$\mathrm{DS}\;\left(\%\right)=\frac{W2-W1}{W1}\times 100$$

9 $$\mathrm{WS}\;\left(\%\right)=\frac{W1-W3}{W1}\times 100$$

#### 4.5.4 Film Thickness

Film thickness (mm) was measured using an electronic digital micrometer (0–25 mm) with a precision level of 0.001 mm. Ten measurements were obtained at random points on the films and the average thickness was calculated.

#### 4.5.5 Color

The film’s color was measured using a CIE Lab calorimeter (CR400, Konica Minolta, Japan), and its coordinates (L*, a*, b*) were determined. The color values were calculated by taking the average of five measurements at various positions for each sample.

#### 4.5.6 Light Transmission, Film Transparency, and Opacity

The transmittance of the films was measured at selected wavelengths (400–800 nm), using a UV–VIS spectrophotometer (SP 3000 nano, Optima, Japan)^104^ with a slight modification. Each edible film treatment was cut into rectangles with an approximate area (0.5 cm × 3 cm) and placed in a quartz cuvette. The transparency was calculated by measuring the film absorbance at 600 nm and according to the method^105^ using the following equation

10 $$\mathrm{transparency}=\frac{A600}{X}$$

where A600 is the absorbance at 600 nm and X is the film thickness (mm). Three replicates of each edible film treatment were tested.

The absorbance spectrum between 400 and 800 nm was recorded, and the opacity was calculated using the equation opacity (AU, nm) = absorbance units × nanometers.^106^

#### 4.5.7 Statistical Analysis

All analyses were carried out in triplicate and results expressed as the average value ± standard deviation (SD). Statistical analyses were performed using one-way analysis of variance, and the significant difference of means was determined using Tukey’s test (SPSS for Windows, Version 20, SPSS Inc., Chicago, USA) at a 95% confidence level (*p* < 0.05).

## Data Availability

The data that support the findings of this study are available on request from the corresponding author.

## References

[1] Puscaselu R., Gutt G., Amariei S. (2020). The use of edible films based on
sodium alginate in meat product packaging: an Eco-Friendly alternative
to conventional plastic materials. Coatings.

[2] Hounsou M., Dabadé D. S., Götz B., Hounhouigan M. H., Honfo F. G., Albrecht A., Dresch L. C., Kreyenschmidt J., Hounhouigan D. J. (2022). Development
and use of food packaging from plant leaves
in developing countries. J. Verbrauch. Lebensm..

[3] Otto S., Strenger M., Maier-Nöth A., Schmid M. (2021). Food packaging and
sustainability – Consumer perception vs. correlated scientific
facts: A review. J. Clean. Prod..

[4] Murrieta-Martínez C. L., Soto-Valdez H., Pacheco-Aguilar R., Torres-Arreola W., Rodríguez-Felix F., Márquez Ríos E. (2018). Edible protein
films: Sources and behavior. Packaging Technology
and Science.

[5] Dehnad D., Mirzaei H., Emam-Djomeh Z., Jafari S. M., Dadashi S. (2014). Thermal and
antimicrobial properties of chitosan-nanocellulose films for extending
shelf life of ground meat. Carbohydr. Polym..

[6] Tajik S., Maghsoudlou Y., Khodaiyan F., Jafari S. M., Ghasemlou M., Aalami M. (2013). Soluble soybean polysaccharide: A new carbohydrate
to make a biodegradable film for sustainable green packaging. Carbohydr. Polym..

[7] Gorrasi G., Viscusi G., Gerardi C., Lamberti E., Giovinazzo G. (2022). Physicochemical
and Antioxidant Properties of White (Fiano cv) and Red (Negroamaro
cv) Grape Pomace Skin Based Films. J. Polym.
Environ..

[8] Maraveas C. (2020). Production
of sustainable and biodegradable polymers from agricultural waste. Polymers.

[9] Jariyasakoolroj P., Leelaphiwat P., Harnkarnsujarit N. (2020). Advances in research and development
of Bioplastic for food packaging. J. Sci. Food
Agric..

[10] Zubair M., Ullah A. (2020). Recent advances in protein derived bionanocomposites for food packaging
applications. Crit. Rev. Food Sci. Nutr..

[11] Jafarzadeh S., Jafari S. M., Salehabadi A., Nafchi A. M., Uthaya
Kumar U. S., Khalil H. P. S. A. (2020). Biodegradable green packaging with
antimicrobial functions based on the bioactive compounds from tropical
plants and their by-products. Trends Food Sci.
Technol..

[12] Otoni C. G., Avena-Bustillos R. J., Olsen C. W., Bilbao-Sáinz C., McHugh T. H. (2016). Mechanical and water barrier properties of isolated
soy protein composite edible films as affected by carvacrol and cinnamaldehyde
micro and nanoemulsions. Food Hydrocoll..

[13] Wang H., Chen K., Cheng J., Jiang L., Yu D., Dai Y., Wang L. (2021). Ultrasound-assisted
three phase partitioning for simultaneous
extraction of oil, protein and polysaccharide from pumpkin seeds. LWT - Food Sci. Technol..

[14] Lalnunthari C., Devi L. M., Badwaik L. S. (2020). Extraction
of protein and pectin
from pumpkin industry by-products and their utilization for developing
edible film. J. Food Sci. Technol..

[15] Rezig L., Chibani F., Chouaibi M., Dalgalarrondo M., Hessini K., Guéguen J., Hamdi S. (2013). Pumpkin (Cucurbita
maxima) seed proteins: sequential extraction processing and fraction
characterization. J. Agric. Food Chem..

[16] Yang F., Huang X., Zhang C., Zhang M., Huang C., Yang H. (2018). Amino acid composition
and nutritional value evaluation of Chinese
chestnut (: Castanea mollissima Blume) and its protein subunit. RSC Adv..

[17] Xu X., Liu H., Duan S., Liu X., Zhang K., Tu J. (2019). A novel pumpkin
seeds protein-pea starch edible film: Mechanical, moisture distribution,
surface hydrophobicity, UV-barrier properties and potential application. Mater. Res. Express.

[18] MAPMDREF . Official Ministry website. 2022. https://www.agriculture.gov.ma.

[19] Sicaire, A.-G. ; Fine, F. ; Quinsac, A. ; Boukroufa, M. ; Rakotomanomana, N. ; Chemat, F. Innovative Techniques and Alternative Solvents for Green Extraction of Proteins from Pulses and Oleaginous Meals as Industrial Sources for Food and Feed. In Green Extraction of Natural Products: Theory and Practice; Chemat, F. , Ed.; Springer, Singapore, 2019; pp 237–256. 10.1007/978-981-13-3810-6\_9.

[20] Baca-Bocanegra B., Nogales-Bueno J., Hernández-Hierro J. M., Heredia F. J. (2021). Optimization
of Protein Extraction of Oenological Interest from Grape Seed Meal
Using Design of Experiments and Response Surface Methodology. Foods.

[21] Sert D., Rohm H., Struck S. (2022). Ultrasound-Assisted Extraction of
Protein from Pumpkin Seed Press Cake: Impact on Protein Yield and
Techno-Functionality. Foods.

[22] Cabral E. M., Poojary M. M., Lund M. N., Curtin J., Fenelon M., Tiwari B. K. (2022). Effect of solvent
composition on the extraction of
proteins from hemp oil processing stream. J.
Sci. Food Agric..

[23] Das M., Devi L. M., Badwaik L. S. (2022). Ultrasound-assisted extraction of
pumpkin seeds protein and its physicochemical and functional characterization. Appl. Food Res..

[24] Langyan S., Yadava P., Khan F. N., Dar Z. A., Singh R., Kumar A. (2022). Sustaining Protein
Nutrition Through Plant-Based Foods. Front.
Nutr..

[25] Prandi B., Di Massimo M., Tedeschi T., Rodríguez-Turienzo L., Rodríguez Ó. (2022). Ultrasound
and Microwave-assisted
Extraction of Proteins from Coffee Green Beans: Effects of Process
Variables on the Protein Integrity. Food Bioprocess
Technol..

[26] Yu X., Gouyo T., Grimi N., Bals O., Vorobiev E. (2016). Ultrasound
enhanced aqueous extraction from rapeseed green biomass for polyphenol
and protein valorization. C. R. Chim..

[27] Wang Z., Zhang L., Zhang X., Zeng M., He Z., Chen J. (2021). Interfacial Rheology and Foaming Properties of Soy Protein and Hydrolysates
under Acid Condition. Food Biophys..

[28] Moosavi M. H., Khani M. R., Shokri B., Hosseini S. M., Shojaee-Aliabadi S., Mirmoghtadaie L. (2020). Modifications
of protein-based films using cold plasma. Int.
J. Biol. Macromol..

[29] Perera K. Y., Prendeville J., Jaiswal A. K., Jaiswal S. (2022). Cold Plasma Technology
in Food Packaging. Coatings.

[30] Das D., Panesar P. S., Saini C. S., Kennedy J. F. (2022). Improvement in properties
of edible film through non-thermal treatments and nanocomposite materials:
A review. Food Packag. Shelf Life.

[31] Oner M. E., Gultekin Subasi B., Ozkan G., Esatbeyoglu T., Capanoglu E. (2023). Efficacy of
cold plasma technology on the constituents
of plant-based food products: Principles, current applications, and
future potentials. Food Res. Int..

[32] Yashwant A. S., Kashyap P., Goksen G. (2023). Recent advances
in the improvement
of protein-based edible films through non-thermal and thermal techniques. Food Biosci..

[33] Liyanage S., Acharya S., Parajuli P., Shamshina J. L., Abidi N. (2021). Production and Surface Modification of Cellulose Bioproducts. Polymers.

[34] Yao S., Li W., Wu Y., Martin G. J. O., Ashokkumar M. (2023). The Impact
of High-Intensity Ultrasound-Assisted Extraction on the Structural
and Functional Properties of Hempseed Protein Isolate (HPI). Foods.

[35] Quintero-Quiroz J., Celis-Torres A., Ciro-Gómez G., Torres J., Corrales-García L., Rojas J. (2022). Physicochemical properties and functional characteristics of ultrasound-assisted
legume-protein isolates: a comparative study. J. Food Sci. Technol..

[36] Byanju B., Rahman M., Hojilla-Evangelista M.
P., Lamsal B. P. (2020). Effect
of high-power sonication pretreatment on extraction and some physicochemical
properties of proteins from chickpea, kidney bean, and soybean. Int. J. Biol. Macromol..

[37] Mu L., Zhao M., Yang B., Zhao H., Cui C., Zhao Q. (2010). Effect of
Ultrasonic Treatment on the Graft Reaction between Soy
Protein Isolate and Gum Acacia and on the Physicochemical Properties
of Conjugates. J. Agric. Food Chem..

[38] Shen L., Pang S., Zhong M., Sun Y., Qayum A., Liu Y., Rashid A., Xu B., Liang Q., Ma H., Ren X. (2023). A comprehensive review
of ultrasonic assisted extraction (UAE) for
bioactive components: Principles, advantages, equipment, and combined
technologies. Ultrason Sonochem..

[39] Narale B. A., Mounika A., Shanmugam A. (2024). Modifications
of physicochemical,
functional, structural, and nutritional properties of a field bean
protein isolate obtained using batch and continuous ultrasound systems. Sustainable Food Technology.

[40] Gao Z., Shen P., Lan Y., Cui L., Ohm J. B., Chen B., Rao J. (2020). Effect of alkaline extraction pH
on structure properties, solubility, and beany flavor of yellow pea
protein isolate. Food Res. Int..

[41] Lam A. C. Y., Can Karaca A., Tyler R. T., Nickerson M. T. (2018). Pea protein
isolates: Structure, extraction, and functionality. Food Rev. Int..

[42] Shilpashree B. G., Arora S., Chawla P., Tomar S. K. (2015). Effect of succinylation
on physicochemical and functional properties of milk protein concentrate. Food Res. Int..

[43] Achouri A., Nail V., Boye J. I. (2012). Sesame protein isolate:
Fractionation,
secondary structure and functional properties. Food Res. Int..

[44] Bucko S., Katona J., Popovic L., Vastag Z., Petrovic L., Vucinic-Vasic M. (2015). Investigation on solubility, interfacial
and emulsifying
properties of pumpkin (Cucurbita pepo) seed protein isolate. LWT.

[45] Chen J., Mu T., Zhang M., Goffin D., Sun H., Ma M., Liu X., Zhang D. (2018). Structure, physicochemical,
and functional properties
of protein isolates and major fractions from cumin (Cuminum cyminum)
seeds. Int. J. Food Prop..

[46] Deng Q., Wang L., Wei F., Xie B., Huang F. H., Huang W., Shi J., Huang Q., Tian B., Xue S. (2011). Functional properties of protein
isolates, globulin and albumin extracted
from Ginkgo biloba seeds. Food Chem..

[47] Du Y., Jiang Y., Zhu X., Xiong H., Shi S., Hu J., Peng H., Zhou Q., Sun W. (2012). Physicochemical and
functional properties of the protein isolate and major fractions prepared
from Akebia trifoliata var. australis seed. Food Chem..

[48] El-Adawy T. A., Rahma E. H., El-Bedawey A. A., Gafar A. F. (2001). Nutritional potential
and functional properties of sweet and bitter lupin seed protein isolates. Food Chem..

[49] Horax R., Hettiarachchy N., Kannan A., Chen P. (2011). Protein extraction
optimization, characterisation, and functionalities of protein isolate
from bitter melon (Momordica charantia) seed. Food Chem..

[50] Vinayashree S., Vasu P. (2021). Biochemical, nutritional
and functional properties of protein isolate
and fractions from pumpkin (Cucurbita moschata var. Kashi Harit) seeds. Food Chem..

[51] Wani A. A., Sogi D. S., Singh P., Shivhare U. S. (2011). Characterization
and functional properties of watermelon (Citrullus lanatus) seed protein
isolates and salt assisted protein concentrates. Food Sci. Biotechnol..

[52] Zhao H., Shen C., Wu Z., Zhang Z., Xu C. (2020). Comparison
of wheat, soybean, rice, and pea protein properties for effective
applications in food products. J. Food Biochem..

[53] Deng Y., Huang L., Zhang C., Xie P., Cheng J., Wang X., Li S. (2019). Physicochemical and
functional properties
of Chinese quince seed protein isolate. Food
Chem..

[54] Ngo N. T. T., Shahidi F. (2021). Functional
properties of protein isolates from camelina
(Camelina sativa (L.) Crantz) and flixweed (sophia, Descurainis sophia
L.) seed meals. Food Prod. Process. Nutr..

[55] Affandi D. R., Praseptiangga D., Nirmala F. S., Amanto B. S., Atmaka W. (2017). Physical and
Chemical Characterization of Greater Yam (Dioscorea Alata) and Jack
Bean (Canavalia Ensiformis) - Based Composite Flour. IOP Conf. Ser.: Mater. Sci. Eng..

[56] Thakur R., Nimbalkar R. (2020). Quinoa and
Chia Seed : Protein Isolates, Properties,
Nutrition and Health benefits. Int. J. Adv.
Res..

[57] Martínez-Flores H. E., Barrera E. S., Garnica-Romo M. G., Penagos C. J. C., Saavedra J. P., Macazaga-Alvarez R. (2006). Functional
characteristics of protein flaxseed concentrate
obtained applying a response surface methodology. J. Food Sci..

[58] Güzel M., Çelik M., Yildirim M. (2019). Effect of pH on Protein Extraction
from Mahaleb Kernels and Functional Properties of Resulting Protein
Concentrate. Int. J. Food Eng..

[59] Ghribi A. M., Gafsi I. M., Blecker C., Danthine S., Attia H., Besbes S. (2015). Effect of drying methods
on physico-chemical and functional
properties of chickpea protein concentrates. J. Food Eng..

[60] Mwasaru M. A., Muhammad K., Bakar J., Man Y. B. C. (1999). Effects of isolation
technique and conditions on the extractability, physicochemical and
functional properties of pigeonpea (Cajanus cajan) and cowpea (Vigna
unguiculata) protein isolates. I. Physicochemical properties. Food Chem..

[61] Feyzi S., Milani E., Golimovahhed Q. A. (2018). Grass Pea
(Lathyrus sativus L.) Protein
Isolate: The Effect of Extraction Optimization and Drying Methods
on the Structure and Functional Properties. Food Hydrocoll..

[62] Ma K. K., Greis M., Lu J., Nolden A. A., McClements D. J., Kinchla A. J. (2022). Functional Performance of Plant Proteins. Foods.

[63] Lam A. C. Y., Can
Karaca A., Tyler R. T., Nickerson M. T. (2018). Pea protein
isolates: Structure, extraction, and functionality. Food Rev. Int..

[64] Schmitt C., Bovetto L., Buczkowski J., De Oliveira Reis G., Pibarot P., Amagliani L., Dombrowski J. (2021). Plant proteins
and their colloidal state. Curr. Opin. Colloid
Interface Sci..

[65] Sim S. Y. J., Srv A., Chiang J. H., Henry C. J. (2021). Plant proteins for
future foods: A roadmap. Foods.

[66] Van
der Ven C., Gruppen H., De Bont D. B. A., Voragen A. G. J. (2002). Correlations
between biochemical characteristics and foam-forming and -stabilizing
ability of whey and casein hydrolysates. J.
Agric. Food Chem..

[67] Akintayo E. T., Oshodi A. A., Esuoso K. O. (1999). Effects
of NaCl, ionic strength and
pH on the foaming and gelation of pigeon pea (Cajanus cajan) protein
concentrates. Food Chem..

[68] Sathe S. K., Deshpande S. S., Salunkhe D. K. (1982). Functional Properties of Lupin Seed
(Lupinus mutabilis) Proteins and Protein Concentrates. J. Food Sci..

[69] Chen Y., Duan Q., Yu L., Xie F. (2021). Thermomechanically
processed chitosan:gelatin films being transparent, mechanically robust
and less hygroscopic. Carbohydr. Polym..

[70] Dahlmann F., Dinu D. F., Jusko P., Lochmann C., Gstir T., Marimuthu A. N., Liedl K. R., Brünken S., Wester R. (2023). Vibrational Predissociation
Spectra of C2N–
and C3N–: Bending and Stretching Vibrations. ChemPhysChem.

[71] Qiao C., Ma X., Zhang J., Yao J. (2017). Molecular interactions in gelatin/chitosan
composite films. Food Chem..

[72] Fu L., Chen S. L., Wang H. F. (2016). Validation
of Spectra and Phase in
Sub-1 cm(−1) Resolution Sum-Frequency Generation Vibrational
Spectroscopy through Internal Heterodyne Phase-Resolved Measurement. J. Phys. Chem. B.

[73] Jeevahan J., Mageshwaran G., Joseph G. B., Raj R. B. D., Kannan R. T. (2017). Various
Strategies for Reducing NOx Emissions of Biodiesel Fuel Used in Conventional
Diesel Engines: A Review. Chem. Eng. Commun..

[74] Lalnunthari C., Devi L. M., Amami E., Badwaik L. S. (2019). Valorisation of
pumpkin seeds and peels into biodegradable packaging films. Food Bioprod. Process..

[75] Li Z., Deng S., Chen J. (2022). Surface Modification via Dielectric
Barrier Discharge Atmospheric Cold Plasma (DBD–ACP): Improved
Functional Properties of Soy Protein Film. Foods.

[76] Ortega M. L. S., Orellana-Palacios J. C., Garcia S. R., Rabanal-Ruiz Y., Moreno A., Hadidi M. (2024). Olive leaf
protein: Extraction optimization,
in vitro digestibility, structural and techno-functional properties. Int. J. Biol. Macromol..

[77] Tan E. S., Ying-Yuan N., Gan C. Y. (2014). A comparative study of physicochemical
characteristics and functionalities of pinto bean protein isolate
(PBPI) against the soybean protein isolate (SPI) after the extraction
optimization. Food Chem..

[78] Coltelli M.-B., Wild F., Bugnicourt E., Cinelli P., Lindner M., Schmid M., Weckel V., Müller K., Rodriguez P., Staebler A. (2016). State
of the Art in
the Development and Properties of Protein-Based Films and Coatings
and Their Applicability to Cellulose Based Products: An Extensive
Review. Coatings.

[79] Kehrer M., Duchoslav J., Hinterreiter A., Mehic A., Stehrer T., Stifter D. (2020). Surface functionalization of polypropylene using a
cold atmospheric pressure plasma jet with gas water mixtures. Surf. Coat. Technol..

[80] Sheikhi Z., Mirmoghtadaie L., Khani M. R., Farhoodi M., Beikzadeh S., Abdolmaleki K., Kazemian-Bazkiaee F., Shokri B., Shojaee-Aliabadi S. (2020). Physicochemical
Characterization of Argon Plasma-Treated Starch Film. J. Agric. Sci. Technol..

[81] Ji H., Dong S., Han F., Li Y., Chen G., Li L., Chen Y. (2018). Effects of Dielectric Barrier Discharge (DBD) Cold
Plasma Treatment on Physicochemical and Functional Properties of Peanut
Protein. Food Bioprocess Technol..

[82] Nazari B., Mohammadifar M. A., Shojaee-Aliabadi S., Feizollahi E., Mirmoghtadaie L. (2018). Effect of
ultrasound treatments on functional properties
and structure of millet protein concentrate. Ultrason. Sonochem..

[83] Sharifian A., Soltanizadeh N., Abbaszadeh R. (2019). Effects of dielectric barrier discharge
plasma on the physicochemical and functional properties of myofibrillar
proteins. Innov. Food Sci. Emerg. Technol..

[84] Kavoosi G., Dadfar S. M. M., Purfard A. M. (2013). Mechanical,
Physical, Antioxidant,
and Antimicrobial Properties of Gelatin Films Incorporated with Thymol
for Potential Use as Nano Wound Dressing. J.
Food Sci..

[85] Sani I. K., Aminoleslami L., Mirtalebi S. S., Sani M. A., Mansouri E., Eghbaljoo H., Jalil A. T., Thanoon R. D., Khodaei S. M., Mohammadi F., Kazemzadeh B. (2023). Cold plasma technology: Applications
in improving edible films and food packaging. Food Packag. Shelf Life.

[86] Sharath
Kumar N., Dar A. H., Dash K. K., Kaur B., Pandey V. K., Singh A., Fayaz U., Shams R., Mukarram S. A., Kovacs B. (2024). Recent advances in cold plasma technology
for modifications of proteins: A comprehensive review. J. of Agri. and Food Res..

[87] Santhosh R., Madhu Babu D., Thakur R., Nath D., Hoque M., Gaikwad K. K., Ahmed J., Sarkar P. (2024). Effect of
atmospheric
cold plasma treatment on structural, thermal, and mechanical properties
of pea protein isolate edible films. Sustain.
Chem. Pharm..

[88] Zhang B., Tan C., Zou F., Sun Y., Shang N., Wu W. (2022). Impacts of
Cold Plasma Technology on Sensory, Nutritional and Safety Quality
of Food: A Review. Foods.

[89] Gholamazad A., Hosseini S., Hosseini S., Ramezan Y., Rahmanabadi A. (2022). Effect of
low-pressure cold plasma on the properties of edible film based on
alginate enriched with Dunaliella salina powder. Plasma Process. Polym..

[90] Liu C., Huang J., Zheng X., Liu S., Lu K., Tang K., Liu J. (2020). Heat sealable soluble
soybean polysaccharide/gelatin
blend edible films for food packaging applications. Food Packag. Shelf Life.

[91] Zhou G. H., Xu X. L., Liu Y. (2010). Preservation technologies
for fresh
meat – A review. Meat Sci..

[92] Balali S., Davachi S. M., Sahraeian R., Shiroud Heidari B., Seyfi J., Hejazi I. (2018). Preparation and Characterization
of Composite Blends Based on Polylactic Acid/Polycaprolactone and
Silk. Biomacromolecules.

[93] Davachi S. M., Shekarabi A. S. (2018). Preparation and characterization
of antibacterial,
eco-friendly edible nanocomposite films containing Salvia macrosiphon
and nanoclay. Int. J. Biol. Macromol..

[94] Davoodi S., Davachi S. M., Ghorbani
Golkhajeh A., Shekarabi A. S., Abbaspourrad A. (2020). Development
and Characterization of Salvia macrosiphon/Chitosan
Edible Films. ACS Sustain. Chem. Eng..

[95] El-Hefian E. A., Nasef M. M., Yahaya A. H. (2012). Preparation and Characterization
of Chitosan/Agar Blended Films: Part 1. Chemical Structure and Morphology. J. Chem..

[96] Chen G. Y., Yang T. L., Wang Y. H., Li S. H., Chen Y. (2023). Properties
enhancement of antimicrobial chitosan-deposited polylactic acid films
via cold plasma treatment. Food Health.

[97] Ding L., Shao L., Bai Y. (2014). Deciphering
the mechanism of corona
discharge treatment of BOPET film. RSC Adv..

[98] Hu Y., Wang Z., Zhang X., Bai X., Li X., Ren D. F. (2021). Development of whey protein isolate/chitosan/microcrystalline
cellulose-based bilayer films using surface-pretreated polyethylene
terephthalate substrate. J. Food Process. Eng..

[99] Bera M. B., Mukherjee R. K. (1989). Solubility,
Emulsifying, and Foaming Properties of
Rice Bran Protein Concentrates. J. Food Sci..

[100] American Association of Cereal Chemists (AACC) . Approved methods of the AACC, 8th ed.; AACC: St. Paul, MN, 1986; Method 74-09.

[101] Sharma L., Singh C., Sharma H. K. (2016). Assessment of functionality
of sesame meal and sesame protein isolate from Indian cultivar. J. Food Meas. Charact..

[102] Lawal O. S., Adebowale K. O., Ogunsanwo B. M., Sosanwo O. A., Bankole S. A. (2005). On the functional
properties of globulin
and albumin protein fractions and flours of African locust bean (Parkia
biglobossa). Food Chem..

[103] Isik I., Yenipazar H., Saygun A., Sahin Yesilcubuk N., Ozkan Zayim E., Catalgil Giz H. (2023). Aloe vera Oil-Added Agar Gelatin
Edible Films for Kashar Cheese Packaging. ACS
Omega.

[104] Fang Y., Tung M. A., Britt I. J., Yada S., Dalgleish D. G. (2002). Tensile and barrier properties of edible films made
from whey proteins. J. Food Sci..

[105] Kurt A., Kahyaoglu T. (2014). Characterization
of a new biodegradable
edible film made from salep glucomannan. Carbohydr.
Polym..

[106] Mali S., Beatriz Karam L., Pereira Ramos L., Victo Ä, Ria Grossmann M. E. (2004). Relationships among the Composition
and Physicochemical Properties of Starches with the Characteristics
of Their Films. J. Agric. Food Chem..

